# Lifestyle interventions and quality of life for women with polycystic ovary syndrome: A systematic review and meta-analysis protocol

**DOI:** 10.1097/MD.0000000000018323

**Published:** 2019-12-16

**Authors:** Romilson de Lima Nunes, Isis Kelly dos Santos, Ricardo Ney Cobucci, Gabriel Soares Pichini, Gustavo Mafaldo Soares, Tecia Maria de Oliveira Maranhão, Paulo Moreira Silva Dantas

**Affiliations:** a Department of Physical Activity of the Federal University of Rio Grande do Norte; b Health Sciences Graduate Program of the Federal University of Rio Grande do Norte; c Biotechnology Postgraduate Program of the Potiguar University of Rio Grande do Norte; d Department of Gynecology and Obstetrics of the Federal University of Rio Grande do Norte, Natal, Brazil.

**Keywords:** exercise intervention, health behavior, nutrition, polycystic ovarian syndrome, quality of life

## Abstract

**Background:**

Polycystic ovary syndrome (PCOS) is one of the most common endocrine disorders in women of reproductive age. PCOS has a significant negative impact on the health-related quality of life (HRQoL) and psychological function of women, of which there are reports of high levels of depression in women with PCOS compared to those without PCOS. However, the evidence surrounding the effects of exercise and/or dietary intervention participation on the HRQoL of women with PCOS is limited. Therefore, our objective is to examine the effects of lifestyle interventions (definition include exercise-only, diet-only, exercise + diet and behavioral or combined) on health-related quality of life or general quality of life in women with PCOS.

**Methods:**

We will conduct an update of systematic review and we will follow the recommendations and guidelines of the Cochrane handbook for systematic reviews and Preferred Reporting Items for Systematic Review and Meta-Analysis Protocol (PRISMA-P). We will search the studies in the following databases: MEDLINE. PubMed, PsychINFO, Embase, SportDiscus, Web of Science, Cochrane Database (via Cochrane library), Cochrane Controlled Register of Trials (CENTRAL), and Google Scholar (advance). Manual search of the reference list of identified works, without language and year restrictions. The process of study selection and data extraction will be performed independently by 2 reviewers, with a third reviewer being responsible for the final decision in case of disagreement between the first two. We will use Egger funnel chart to evaluate possible publication biases, in addition, when possible we will perform a subgroup/meta-regression analysis. The strength of the evidence will be assessed according to the Grading of Recommendations Assessment, Development, and Evaluation (GRADE).

**Protocol registry:**

PROSPERO number: CRD42019124176.

## 1 Introduction

Polycystic ovary syndrome (PCOS) is a common endocrine dysfunction among women of reproductive age, with a prevalence of 4% to 12% but its etiology remains unknown.^[1]^ The symptoms of PCOS such as hirsutism, menstrual irregularity and infertility cause associated morbidities that generate negative consequences in the psychological and interpersonal functioning of individuals.^[2]^

For many women, the consequences of PCOS can lead to stress in their personal and social.^[3]^ The psychological impact of PCOS is substantial, with incidence of depression and anxiety varying from 28% to 64%.^[4]^ The literature showed symptoms of PCOS is a major cause of psychological morbidity and has a negative impact on women's HRQoL.^[1–5]^ Faced with this problem, International guidelines suggest a special look at the psychological factors and quality of life of women with PCOS, in which a screening of possible symptoms of depression and anxiety need to be tracked during consultations.^[6]^

According to the World Health Organization (WHO) the concept of Quality of Life (QoL) over time has been interpreted in several different ways, since initially the concept was related to purchasing power and its ways of providing different life conditions.^[7]^ However, with the increase in life expectancy, advances in science and survival of patients with chronic diseases, the concept of quality of life has been expanded and modified, and now there is a relationship with the health condition.^[8]^ Thus, health-related quality of life (HRQoL) refers to the individual's perception of the condition of their own health, well-being and their perception of the consequences and treatments related to any disease that may affect their life condition.^[9]^

Therefore, studying the quality of life related to the health of people with instruments such as questionnaires or scales allows health professionals to assess the impact of syndromes such as PCOS.^[10]^ In this sense, the guidelines recommend as a form of no pharmacologic treatment for women with PCOS, the insertion of exercises and a diet with caloric restriction as crucial elements, in order to reduce the consequences of the symptoms of PCOS on the health and consequently on the quality of life of women.^[11]^

Lifestyle interventions (definition include exercise only, diet only, exercise + diet and behavioral or combined) is a first-line non-pharmacological treatment for PCOS; Thus, there are a large number of studies evaluating the impact of exercise alone or in conjunction with diet (lifestyle intervention) on the improvement of reproductive, anthropometric and metabolic factors in women with PCOS showing consistent results of these interventions on a variety of factors.^[12–18]^

Some of these systematic reviews pointed out, addressing the quality of life result as secondary, that is, the searches were not specific about this type of result, so, according to this analysis, there is a need to deepen this subject once Although the published meta-analyzes show the efficacy of this type of intervention in the risk factors for PCOS, the evidence surrounding the effects of this lifestyle intervention on the quality of life and consequently on the mental health of women with PCOS is found limited.^[13,14,16]^

In the literature, only one systematic review published in 2015 addressed this specific theme.^[19]^ Thus, the current systematic review proposal aims to update it in order to discuss the evidence and describe the effects of lifestyle modification on these parameters, as there are several recently published clinical trials on the subject.^[20]^

In this regard, a new systematic review on the effects of lifestyle intervention on quality-of-life aspects of women with PCOS with different phenotypes and ages will help minimize bias and provide new evidence on the benefits of non-drug treatment programs. Overdue to the increasing prevalence of women with PCOS presenting impairment in psychological functioning, an alert has been aroused in the health service.^[21]^ Thus, our primary objective with this systematic review update is to examine the effects of lifestyle interventions on health-related quality of life of life in women with PCOS. The secondary specific aims are to synthesize available evidence for the intervention implementation factors, such as type of exercise, intensity, frequency, and adherence as well as the quality of life factors such as physical aspects, pain, general health, vitality, social aspects, emotional, and mental.

## 2 Methods/design

### 2.1 Protocol and registration

This systematic review protocol will follow the recommendations and guidelines of the Cochrane handbook for systematic reviews and Preferred Reporting Items for Systematic Review and Meta-Analysis Protocol (PRISMA-P).^[22]^ This protocol was registered in Prospero (registration number: CRD42019124176).

### 2.2 Types of studies

We will include randomized controlled trials (RCT's) and controlled clinical trials (CCT's) with no restriction of language and year of publication.

### 2.3 Types of participants

Studies involving women (aged 18 and older) diagnosed with polycystic ovary syndrome (PCOS) based on Rotterdam criteria, National Institute of Health criteria (NIH) and Androgen Excess PCOS Society criteria.

### 2.4 Types of interventions

We will include all studies that only report on lifestyle intervention (exercise and diet), compared with a control group (no treatment) in regard to the health-related quality of life for women with PCOS. Thus, interventions may include aerobic (walking and / or running), resistance exercise training (strength) and combined programs (aerobic and resistance), dietary therapy or dietary intervention with acute or chronic duration.

### 2.5 Types of outcome measures

#### 2.5.1 Primary outcomes:

We will include studies that address changes in HRQoL specific disease scores, as defined in the studies, measured through questionnaires or validated scales. For example, the specific disease of: Polycystic Ovary Syndrome Questionnaire (PCOSQ), List of Symptoms 90 (SCL-90-R). HRQoL, such as: EuroQol (EQ-5D), Short Form-36 (SF-36), WHOQOL-100 (World Health Organization Quality of Life Assessment) and / or the abridged WHOQOL BREAF and General Health questionnaire (GHQ). The results related to domains such as “well-being”, “self-esteem”, “stress”, “psychological well-being” or “mental health” will be included, since these are factors that are related to general QoL.

#### 2.5.2 Secondary outcomes

Adherence to exercise and dietary; Duration, frequency, intensity and type of exercise; Body Mass Index (BMI); waist and hip circumference, body fat (%) and hirsutism (Ferriman and Gallwey score).

#### 2.5.3 Search methods for identification of studies:

The studies will be identified in the following electronic databases: PubMed, PsychINFO, Embase (via Ovid), SportDiscus, Web of Science, Cochrane Database (via Cochrane library), Cochrane Controlled Register of Trials (CENTRAL) and Google Scholar (*advance*).

Search strategies in the literature will be developed using key words indexed in the Medical Subject Headings (MeSH) and free text terms related to the key concepts of lifestyle intervention (exercise training + diet), health-related quality of life and polycystic ovary syndrome. In addition, systematic reviews, reference lists of papers, conference abstracts, grey literature and trial registers (e.g., ClinicalTrials.gov) will be searched for additional studies; an experienced information specialist librarian will perform all literature searches. This search strategy is described in Table 1.

**Table 1:**
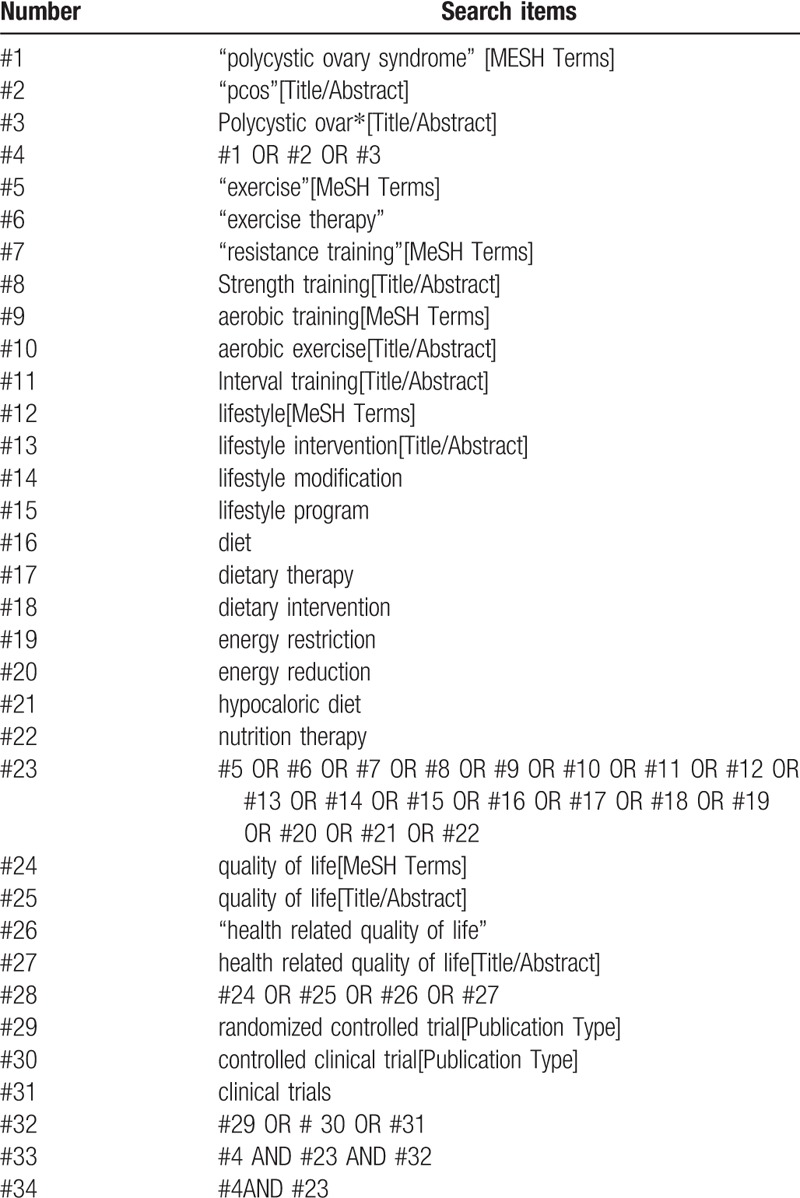
PubMed search strategy.

### 2.6 Data collection and analysis

#### 2.6.1 Selection of studies

**Figure 1:**
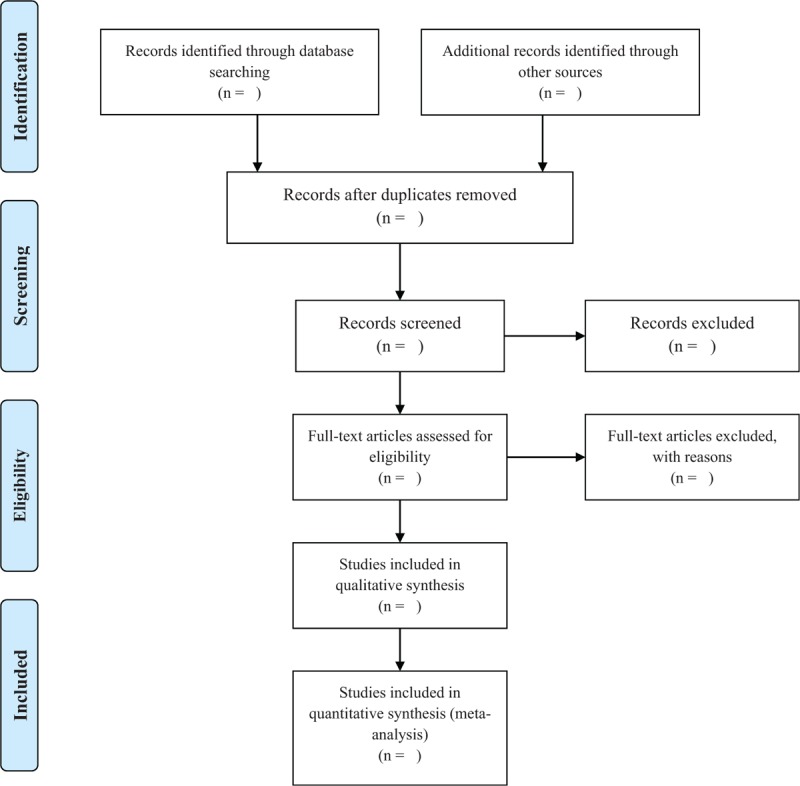
Flow diagram of study process. ^∗^This strategy search will be modified as required for the other databases.

The studies identified will be stored in reference management software (EndNote - Thompson Reuters, CA). All studies identified through search strategies (titles and abstracts) will be independently filtered by two authors (RLN and IKS) based on the inclusion criteria described above (Level 1). Potentially eligible studies will be re-evaluated by reading the full text, as well as studies that present insufficient information for decision making from reading the title (Level 2). In situations of possible disagreements in the evaluations the opinion of a third review author (RNC) will be requested. The process of selecting the studies will be presented with a flowchart and will be reported according to the PRISMA.^[23]^ We will used the Covidence (Covidence systematic review software, Veritas Health Innovation, Melbourne, Australia) for the removal of possible duplicates, screening citations at title and abstract (Level 1), full text (Level 2) and extracting data. The selection process will follow PRISMA flow diagram (Fig. 1).

#### 2.6.2 Data extraction and management

The process of extracting the data from the possible included studies will be through modified Cochrane Data collection form to ensure that all key findings are included accurately in the review. We will collect information concerning the general information (authors, source of funding); eligibility (design of study, setting); participants (definition of PCOS, age; BMI range, eligibility criteria, treatments), interventions (description, time) and data relating to the outcomes specified above. An author (IKS) will extract the data independently, a second author (RLN) will analyze the accuracy and consistency of all extractions and any discrepancy will be analyzed by a third author (RNC). The following data will be extracted from the original studies in Table 2.

**Table 2:**
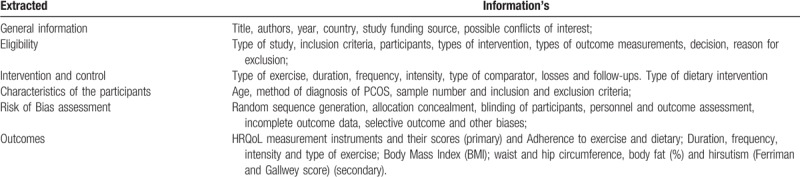
Data form extracted information's.

#### 2.6.3 Assessment of risk of bias in included studies

Two authors (RLN and IKS) will independently evaluate the methodological quality of the studies according to the Cochrane Risk of Bias tool 2.0 (RoB 2.0).^[24]^ All the results will be compared and the discrepancies will be discussed with the presence of a third review author (PMSD) to resolve disagreements. We will use the Cohen k calculation to assess agreement among reviewers.^[25]^

### 2.7 Data synthesis

The results of the systematic review will be written in a structured manner in regard to the type of intervention, characteristics of the target population, type of outcome. After the data extraction, the reviewers will determine if there is a possibility of performing a meta-analysis, by considering if the heterogeneity is moderate or strong, as assessed by *I*^2^ (25%–50%, moderate heterogeneity and> 50% strong heterogeneity), the random effects model will be used for analysis. Means and standard deviations (SD) of measures will be used to compare the effect size of each parameter of HRQoL measurement to allow the creation of forest plots. In addition, when possible we will use Egger funnel chart to evaluate possible publication biases. The data will be entered into the Review Manager software (RevMan 5.2).

### 2.8 Sensitivity analysis

All the studies will be included regardless of their risk of bias, but we will conduct a sensitivity analysis to determine the possible effect of excluding studies with higher risk.

### 2.9 Quality of evidence

The strength of the body of evidence will be assessed according to Grading of Recommendations Assessment, Development and Evaluation (GRADE)^[26]^ using GRADE PRO software (https://gdt.gradepro.org). The GRADE is characterized as a comprehensive instrument in the process of evidence assessment quality of evidence at 4 levels: high, moderate, low, very low and analyze each of the following domains: randomization process, missing outcome data, measurement of the outcome and selection of the reported result. Two reviewers (GMS, PMSD) will evaluate the quality of evidence using GRADE.

### 2.10 Analysis of subgroups

The subgroup analyze will be performed:

(1) Studies with different intensity;
(2) Studies specifically examining different types exercise interventions;
(3) Studies with different dimensions of the HRQoL (physical, psychological, and social well-being) and QoL;

## 3 Discussion

Previous systematic reviews have shown the existence of consistent data on the effects of exercise on HRQoL in the adult population with certain types of disease.^[27–29]^ However, few studies in the literature evaluate the effect of physical exercise or interventions for lifestyle changes on the quality of life of women with PCOS.^[30,31]^

Prescribed to optimize the treatment of women with PCOS, physical exercise has been shown to improve a number of factors and outcomes related to the health of this population, increasing values in regard to ovulation rates, menstrual regularity, cardiorespiratory fitness, and reduction of mental disorders, while decreasing waist circumference and body fat.^[32]^ Thus, guidelines of clinical practice suggest that physical exercises are a positive point for the management of non-pharmacological treatment of PCOS.^[33]^ However, due to the lack of data on the type of exercise, intensity or duration that produces benefits on the quality of life in women with PCOS means that there is not enough evidence on the efficacy of physical activity.

Given the importance of quality of life for the health of women with PCOS, a more detailed and comprehensive view on the effect of exercise is needed. This protocol provides a structured, planned and clear procedure to maximize the extraction of relevant information and provide synthesized information. The results of this systematic review may be of interest to researchers, formulators and health professionals, providing knowledge as the basis for developing effective action plans in this field of knowledge.

## Acknowledgments

The Coordenação de Aperfeiçoamento de Pessoal de Nível Superior/Conselho Nacional de Desenvolvimento Científico e Tecnológico (CAPES/CNPQ) – Higher Education Personnel Improvement Coordination – for PhD scholarship.

## Author contributions

**Conceptualization:** Romilson de Lima Nunes; Isis Kelly dos Santos and Paulo Moreira Silva Dantas.

**Data curation:** Romilson de Lima Nunes, Isis Kelly dos Santos and Ricardo Ney Cobucci.

**Formal analyses:** Romilson de Lima Nunes, Isis Kelly dos Santos and Gabriel Soares Pichini.

**Investigation:** Romilson de Lima Nunes, Isis Kelly dos Santos and Gabriel Soares Pichini.

**Methodology:** Romilson de Lima Nunes, Isis Kelly dos Santos, Tecia Maria de Oliveira Maranhão and Gustavo Mafaldo Soares.

**Supervision:** Tecia Maria de Oliveira Maranhão and Paulo Moreira Silva Dantas.

**Writing – original draft:** Romilson de Lima Nunes, Isis Kelly dos Santos, Ricardo Ney Cobucci and Paulo Moreira Silva Dantas.

**Writing – review and editing:** Romilson de Lima Nunes, Isis Kelly dos Santos, Ricardo Ney Cobucci and Paulo Moreira Silva Dantas.

Isis Kelly Santos orcid: 0000-0001-7615-416X.
